# Dietary Fiber and Metabolic Syndrome: A Meta-Analysis and Review of Related Mechanisms

**DOI:** 10.3390/nu10010024

**Published:** 2017-12-26

**Authors:** Jia-Ping Chen, Guo-Chong Chen, Xiao-Ping Wang, Liqiang Qin, Yanjie Bai

**Affiliations:** Department of Nutrition and Food Hygiene, School of Public Health, Soochow University, 199 Ren’ai Road, Dushu Lake Higher Education District, Suzhou 215123, China; primajiaping@126.com (J.-P.C.); lsguorong@126.com (G.-C.C.); wxplm@suda.edu.cn (X.-P.W.)

**Keywords:** dietary fiber, metabolic syndrome, meta-analysis, mechanisms

## Abstract

(1) Background: Dietary fiber intake may provide beneficial effects on the components of metabolic syndrome (MetS); however, observational studies reported inconsistent results for the relationship between dietary fiber intake and MetS risk. We conducted a meta-analysis to quantify previous observational studies and a narrative review to summarize mechanisms involved in the potential relationship. (2) Methods: The literature was searched on PubMed and Web of Science until 28 November 2017. A random-effects model was used to calculate the summary risk estimates. Eleven cross-sectional studies and three cohort studies were included in the meta-analysis. Results from the original studies were reported as odds ratios (ORs) or relative ratios (RRs) of the MetS associated with different levels of dietary fiber intake, and the ORs/RRs comparing the highest with lowest categories of the intake were pooled. (3) Results: For the cross-sectional studies, the pooled OR was 0.70 (95% confidence interval (CI): 0.61–0.82) with evidence of high heterogeneity (*I*^2^ = 74.4%, *p* < 0.001) and publication bias (*p* for Egger’s test < 0.001). After removing four studies, results remained significant (OR = 0.67, 95% CI: 0.58–0.78) and the heterogeneity was largely reduced (*I*^2^ = 32.4%, *p* = 0.181). For the cohort studies, the pooled RR was 0.86 (95% CI: 0.70–1.06). (4) Conclusion: Although the meta-analysis suggests an inverse association between dietary fiber intake and risk of MetS, and the association was supported by a wide range of mechanism studies, the findings are limited by insufficient cohort data. More prospective studies are needed to further verify the association between dietary fiber intake and the risk of MetS.

## 1. Introduction

Metabolic syndrome (MetS) is a cluster of symptoms that increases the risks for various chronic disease including cardiovascular disease (CVD) and type 2 diabetes mellitus (T2DM) [1,2]. The main features of MetS include abdominal obesity, high blood pressure, hyperglycemia/insulin resistance, and dyslipidemia [3,4]. The most commonly used criteria for diagnosis of MetS are the National Cholesterol Education Program Adult Treatment Panel III (NCEP ATP-III) and the International Diabetes Federation (IDF) [5,6], both of which include fasting plasma glucose, blood pressure, triglycerides (TG), high-density lipoprotein cholesterol (HDL-C), and body fat (waist circumference).

MetS has become a global public health issue. Its prevalence has been estimated to vary between 20–27% in adults from developing countries [7,8,9,10], and even higher in developed nations [11,12,13]. According to the National Health and Nutrition Examination Survey in the U.S., the overall prevalence of MetS increased from 32.9% in 2003–2004 to 34.7% in 2011–2012 [11]. These estimates illustrate the need to control and prevent MetS. Dietary and lifestyle modifications are among the most promising and economically efficient approaches in reducing a wide range of non-communicable chronic diseases, including MetS [14].

Dietary fibers, as defined by the American Association of Cereal Chemists International, are the “edible parts of plants or analogous carbohydrates that are resistant to digestion and absorption in the human small intestine with complete or partial fermentation in the large intestine” [15]. Increasing total dietary fiber has been shown to reduce body fat [16], improve glycemic response [17], as well as reduce blood pressure [18], TG and low-density lipoprotein cholesterol (LDL-C) [19,20]. However, the reported relationship between dietary fiber intake and MetS risk has not been consistent [21]. Therefore, a meta-analysis was performed to summarize published observational studies on the relationship of dietary fiber intake and the risk of MetS. We also reviewed multiple potential mechanisms involved in this possible relation.

## 2. Methods

### 2.1. Literature Search

The Preferred Reporting Items for Systematic Reviews and Meta-Analysis (PRISMA) guidelines were followed in the report of this meta-analysis [22]. Literature search was first conducted on the PubMed and Web of Science databases on 13 January 2017. We conducted the comprehensive literature search on 28 November 2017 by using the following search terms as free texts: (fiber OR fibre OR food component) and (metabolic syndrome OR insulin resistance syndrome) and (cohort OR prospective OR follow-up OR incidence OR cross-sectional OR case-control). Besides, the MeSH terms of “dietary fiber”, “food”, “metabolic syndrome”, “insulin resistance”, “cohort studies”, “longitudinal studies”, “incidence”, “case-control studies”, “cross-sectional studies” were also searched in PubMed. Results from Web of Science were refined by excluding those reports that published as editorials, letters, meetings, books or reviews. Details of the search strategies are provided in Appendix A (Table A1). Additional references from retrieved full publications were also carefully reviewed.

### 2.2. Study Selection

Studies were selected based on the following criteria: (1) The study design was cross-sectional, case-control, or cohort; (2) the exposure of interest was dietary fiber intake; (3) the outcome of interest was MetS; (4) age of the participants was ≥18 years; and (5) odds ratio (OR) or relative risk (RR) with corresponding 95% confidence interval (CI) for the highest versus the lowest dietary fiber intake related to MetS were available. Studies in which all participants were adolescents or patients with cancer were excluded. When one population was reported in several publications, the publications with smaller sample size were excluded to avoid data duplication.

### 2.3. Data Extraction

The following data were extracted from each study: Name of the first author, publication year, study location, length of follow-up (for cohort studies), type of fiber, sex and age of participants, number of participants, comparison of dietary fiber intake, methods of MetS diagnosis and dietary assessment, variables adjusted for in the analysis, and the OR/RR of MetS and corresponding 95% CIs for each category of dietary fiber intake. The most fully adjusted OR/RR was chosen when several estimates for the same exposure were reported with different levels of adjustments.

### 2.4. Quality Assessment

The methodological quality of the included cross-sectional studies was assessed using an 11-item checklist that was recommended by Agency for Healthcare Research and Quality (AHRQ). Article quality was assessed as follows: Low quality = 0–3; moderate quality = 4–7; and high quality = 8–11 [23]. The quality of the included cohort studies was assessed using the Newcastle-Ottawa Scale (NOS), which was recognized as a good study quality assessment tool for cohort studies. Rating criteria for the NOS were as follows: Low quality = 0–5; medium quality = 6–7; and high quality = 8–9 [24].

### 2.5. Statistical Analysis

The statistical analysis was performed with Stata/MP version 14.1 (StataCorp, College Station, TX, USA). A random-effects model was applied to combine risk estimates of MetS for the highest compared with the lowest category of fiber intake. Heterogeneity test was performed using the *I*^2^ and Q statistics. The *I*² statistic describes the percentage of variation across studies that is due to heterogeneity rather than chance [25]. *I*^2^ < 25.0% was considered as little or no heterogeneity, 25–50% was considered as moderate heterogeneity, and >50% suggested high heterogeneity, respectively. For the Q statistic, *p* < 0.1 was considered statistically significant [26]. Franco et al. separately reported results for soluble and insoluble fiber [27]. The results were combined with the inverse variance weight, and the pooled OR was used in the meta-analysis. Cabello-Saavedra et al. [28] reported results based on both NCEP ATP-III and IDF for MetS diagnostic criteria. For this study, we analyzed the results based on the ATP-III in the primary meta-analysis and used IDF-based estimate in a sensitivity analysis. Both OR values from ATP-III and IDF were used in subgroup analysis stratified by MetS assessment. OR values from the study of Fujii et al. [29] and de Oliveira et al. [30] were not included in the subgroup analysis because the study used neither ATP-III nor IDF criteria. Results for men and women in the study of Kouki et al. [31] were treated as two samples. In the studies of Fujii et al. [29] and Kouki et al. [31], ORs and 95% CIs of MetS were reported for continuous fiber intake (e.g., for 1 g/1000 kcal increase). We converted the estimates corresponding to the reported mean fiber intake in the studies, and used the converted estimates in the high vs. low analysis so that individual studies were assigned with statistically reasonable weight. Subgroup and sensitivity analyses were conducted to investigate potential sources of heterogeneity. Publication bias was investigated by funnel plots and Egger’s test [32].

## 3. Results

### 3.1. Study Characteristics

**Figure 1:**
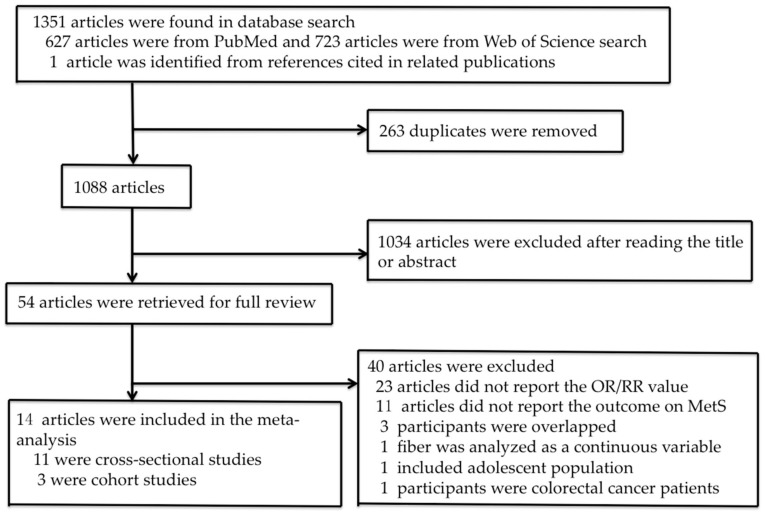
Flow chart of study selection.

The process of study selection is shown in Figure 1. Fifty-four publications were identified for the full-text review. Twenty-three reports were excluded due to the absence of reported RR or OR values and eleven were excluded because study outcome was not reported on MetS. We further excluded two cohort studies and one cross-sectional study because the participants overlapped with the participants in other studies with larger sample sizes [33,34,35]. Other publications were excluded because fiber was analyzed as a continuous variable [36], all participants were adolescent [37] or colorectal cancer patients who were likely to have completely changed their diets due to cancer diagnosis [38]. Finally, fourteen studies were included in the meta-analysis, eleven of which were cross-sectional studies and three were cohort studies. The quality scores of the selected cross-sectional and cohort studies are presented in Table 1 and Table 2, respectively. Eight cross-sectional studies and two cohort studies were of high quality, three cross-sectional studies and one cohort study were of moderate quality.

Detailed characteristics of the cross-sectional studies are presented in Table 1. These cross-sectional studies were carried out in Brazil (*N* = 3), Spain (*N* = 2), Finland (*N* = 1), Italy (*N* = 1), the U.S. (*N* = 2), Japan (*N* = 1) and Iran (*N* = 1). Sample size varied from 175 to 10,473, totaling 26,403 subjects. All cross-sectional studies included both men and women. Eight studies did not specify the type of dietary fiber [28,30,31,39,40,41,42,43], one reported specified fiber types and total fiber [29], Franco et al. [27] reported on soluble and insoluble fiber and Steemburgo et al. [44] reported on soluble fiber and total dietary fiber. Fujii et al. reported total fiber intake as well as fiber from different botanical sources [29]. Criteria for diagnosing MetS were different among the studies. Six studies used the NCEP ATP-III criteria [27,31,40,41,42,43], two studies used the IDF criteria [29,44], one used both [28], one used the modified NCEP ATP-III criteria [30] and one study used harmonized definition [29]. Methods for diet assessment were also different. Two studies used 24 h dietary recalls [30,43], three studies used 3- or 4-day food records [31,39,44], five studies used food frequency questionnaires (FFQ) [27,28,40,41] and one study used a brief diet history questionnaire [29].

Detailed study characteristics for the cohort studies are shown in Table 2. One U.S. study involved 4192 black and white participants from the Coronary Artery Risk Factor Development in Young Adult (CARDIA) with a 15-year follow-up [47]. The other two studies were carried out in Iran [45,46]. The lipid glucose study of Hosseinpour-Niazi et al. [45] involved 1582 participants from Tehran, and the duration of follow-up was three years, whereas Noori et al. [46] followed up 160 renal transplant patients for one year. All studies used the NCEP ATP-III criteria for MetS diagnosis. To assess dietary intake, two studies used FFQ administered by trained dietitians [45,46], and one applied dietary history [47].

### 3.2. Meta-Analysis of Cross-Sectional Studies

**Figure 2:**
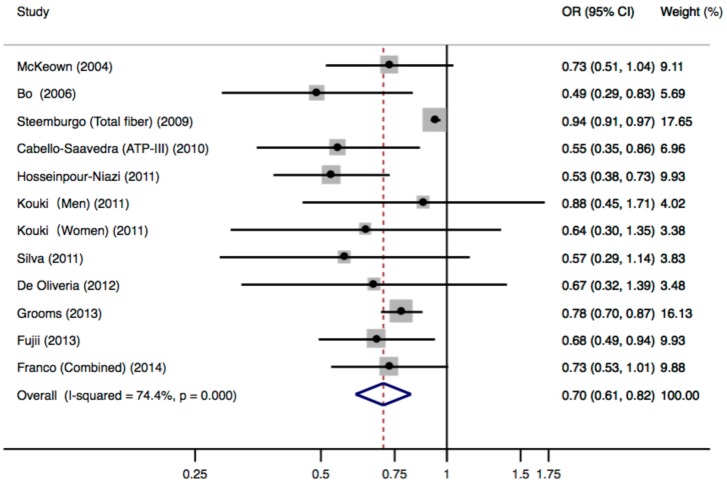
Meta-analysis of fiber intake (comparing the highest with the lowest fiber intake category) and risk of metabolic syndrome for 12 independent samples from 11 cross-sectional studies.

Figure 2 shows the multivariable-adjusted ORs for individual studies and all studies combined for the highest versus lowest categories of dietary fiber intake. The meta-analysis of all cross-sectional studies showed that subjects in the highest category of dietary fiber intake had a significantly decreased risk for MetS, compared with those in the lowest category. The pooled OR was 0.70 (95% CI: 0.61–0.82) and high heterogeneity was observed (*p* < 0.001, *I*^2^ = 74.4%). Two risk estimates were available in the study of Cabello-Saavedra et al. [28] based on different MetS diagnostic criteria. When the estimate based on the IDF criteria was included in the analysis, the pooled OR was 0.71 (95% CI: 0.61–0.83), with similarly high heterogeneity (*p* < 0.001, *I*^2^ = 73.4%).

Sensitivity analysis was performed to investigate the source of heterogeneity. After removing four studies [29,31,39,44], heterogeneity significantly decreased (*p* = 0.181, *I*^2^ = 32.4%), and the pooled OR was 0.67 (95% CI: 0.58–0.78), suggesting that the heterogeneity could be attributed to the four studies removed. When comparing with the subjects in other studies, the four studies have included heterogeneous subjects, such as T2DM patients [29,39,44] and elderly population [31].

Pre-defined subgroup analyses were conducted according to study region, MetS assessment criteria, and number of cases (Table 3). Results were generally consistent with moderate to high heterogeneity.

### 3.3. Meta-Analysis of Cohort Studies

**Figure 3:**
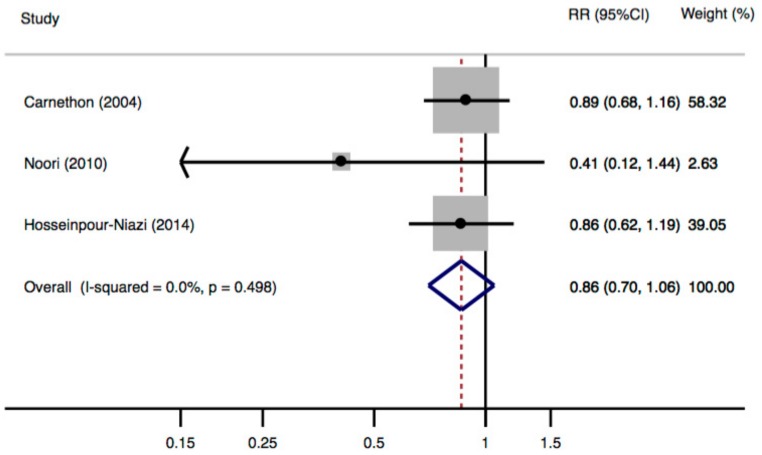
Meta-analysis of fiber intake (comparing the highest with the lowest fiber intake category) and the risk of metabolic syndrome for 3 independent samples from 3 cohort studies.

A meta-analysis of cohort studies suggested a non-significant inverse association between dietary fiber intake and risk of MetS, with a pooled RR of 0.86 (95% CI: 0.70–1.06) when comparing the highest with the lowest category of fiber intake (Figure 3). No heterogeneity was observed (*p* = 0.498, *I*^2^ = 0.0%), and Egger’s test showed no evidence of publication bias (*p* for Egger’s test = 0.031).

## 4. Discussion

### 4.1. Discussion on the Meta-Analysis

This meta-analysis summarized cross-sectional and cohort studies, and showed a possible inverse association between dietary fiber intake and risk for MetS. When considering cross-sectional studies only, consistent results were observed in subgroup analysis regarding region, MetS assessment criteria, and number of cases.

When interpreting the results of this meta-analysis, several factors deserve to be considered. First, age of the participants in the original study may have impacts on the results. The effect of dietary fiber on MetS is suggested to be more pronounced in adolescents [37] and weaker in elderly population (aged 55 years and older) [21]. In this meta-analysis, after removing four studies [28,31,39,44] that only reported results among older populations, the summary estimates from cross-sectional studies remained similar (OR = 0.70, 95% CI: 0.62–0.79) but heterogeneity decreased to 20.8%. Second, the association appeared to be stronger when the difference between the highest category and the lowest category of dietary fiber intake was larger. When comparing fiber intake of 22.5–147.6 g/day versus 0.0–8.1 g/day and 20.3 g/day versus 16.3 g/day, MetS risk reduced by 22% and 6%, respectively [43,44]. Third, different types of fiber may act differently in the prevention of MetS [48]. The effects of dietary fiber differ significantly depending on the botanical source and the processing technologies [49,50]. However, these factors were not taken into account in the original studies. Finally, the criteria for MetS assessment may affect the results and should be considered carefully. Pooling studies that used the ATP-III showed a significant inverse association between dietary fiber intake and risk of MetS.

This meta-analysis also suffers a number of other limitations. Included studies are mostly cross-sectional studies that are prone to various biases. Moreover, T2DM and renal transplant patients were included in some of the original studies [29,31,39,44]. Their health status may influence the effect of dietary fiber on MetS. Furthermore, the meta-analysis showed a significant publication bias. In addition, although a wide range of potential confounding factors have been adjusted for in the original studies, some important confounding factors such as physical activity, smoking and alcohol drinking were not well controlled for in some studies. Therefore, residual confounding by other lifestyle factors cannot be excluded.

During the submission of our work, a meta-analysis evaluating the association between dietary fiber intake and MetS was published by Wei et al. [51]. The study suggests a decreased risk of MetS with increasing dietary fiber intake. One limitation of that meta-analysis is that Wei et al. combined cross-sectional and cohort studies. The included cross-sectional and cohort studies varied substantially in the degree of being subject to study bias and the concluded inverse association based on a combination of these studies is not prudent. In addition, studies by Kouki et al. [31] and Fujii et al. [29] reported fiber as a continuous variable. The risk estimates should be converted for comparing the high vs. low fiber intake, and otherwise, the weight in meta-analysis will be unreasonably assigned. Furthermore, the study by de Oliveira et al. [30] and Kouki et al. [31] were missed by the previous meta-analysis. Overall, the conclusions of our and previous meta-analyses are different, and our meta-analysis suggests that the current evidence is insufficient to support a protective effect of dietary fiber on MetS and more high-quality prospective studies are still needed.

### 4.2. Mechanisms Involved in Dietary Fiber Consumption and MetS

The association between dietary fiber intake and individual components of MetS has been extensively investigated and supported in both observation and intervention studies. Potential mechanisms for health benefits of dietary fiber on MetS are summarized below.

#### 4.2.1. Dietary Fiber and Obesity

Observation studies have constantly highlighted the inverse association between dietary fiber consumption and increase in body weight, BMI or waist circumference [52,53,54,55,56,57,58]. Dietary fiber was also suggested to promote weight loss in obese or overweight individuals and prevent their weight regain [59,60,61,62]. Epidemiological evidence of dietary fiber consumption and obesity or weight regulation has been well reviewed [63,64,65,66]. The mechanisms of dietary fiber on obesity are suggested to be related to energy dilution [66], reduction in nutrients absorption rate [67], appetite suppression [68,69,70], regulation of energy homeostasis [71,72,73], and alternation of gut microbial [74]. Dietary fiber consumption level, physicochemical properties (e.g., solubility and viscosity) [75,76,77,78], fermentability [75] and molecular structure [79] may result in difference in weight regulation.

#### 4.2.2. Dietary Fiber and Insulin Resistance

Consumption of dietary fiber is shown to lower the risk of T2DM in observation studies [80] and meta-analyses [52]. Improved insulin resistance and glucose tolerance in T2DM patients or impaired glucose tolerance subjects was also observed in intervention studies [81,82]. The beneficial effects of dietary fiber on insulin resistance attributes to increasing food glycemic index (GI) [83,84], reducing the risk of obesity [73,85], improving subjects’ glucose homeostasis [71,86], regulating hormonal responses [87], modulating inflammatory cytokines [73,87,88], and altering gut microbiota [74]. The effect of dietary fiber on GI is related to its physicochemical properties including particle size, amount and type of fiber, viscosity, amylose and amylopectin content, delaying gastric emptying time, and reducing glucose absorption [84,89,90,91].

#### 4.2.3. Dietary Fiber and Dyslipidemia

Hypocholesterolemic property of soluble fiber is consistently observed in observation studies [17,92,93] and meta-analyses of randomized controlled trials [20,94,95]. Viscosity of fiber plays a major role in the cholesterol lowering effect [96,97,98]. Soluble fiber of high viscosity reduces plasma cholesterol to a greater extent than insoluble fiber or very low viscosity fiber [99,100]. The cholesterol lowering effect of soluble dietary fiber may attribute to increased fecal bile salts excretion, reduced glycemic response of food, and fermentation products of soluble dietary fiber [101]. Cholesterol lowering effect of insoluble fiber is observed in some studies and the mechanism is attributed to promotion of satiation and satiety [102]. Despite the positive effect on blood cholesterol, effect of fiber on reducing TG or increasing HDL-C is not conclusive [86]. Dietary fiber’s hypotriglyceridemic effects is postulated due to the delayed and reduced absorption of TG and sugars from the small intestine, modulation of fatty acid synthase activity, decreasing the GI and its impact on homeostasis and insulin secretion [86].

#### 4.2.4. Dietary Fiber and Hypertension

An inverse relationship between dietary fiber intake and blood pressure has been observed in several population studies [103,104,105]. Meta-analyses of randomized controlled trials demonstrated that dietary fiber supplementation or intervention of high-fiber diet may result in statistically significant decrease in diastolic blood pressure [106,107]. Mechanisms behind the effects of dietary fiber on blood pressure reduction are still unclear. Increasing dietary fiber may lower the risk of hypertension by controlling the risk factors, such as improving insulin resistance and reducing LDL-C [48,108]. High intake of fiber could also modify gut microbiota populations and increase the abundance of acetate-producing bacteria.

## 5. Conclusions

Our meta-analysis shows that dietary fiber intake is inversely associated with the risk of MetS and the association is supported by a wide range of mechanism studies. However, the findings are limited by scant cohort data; thus, no definitive conclusion could currently be drawn. The evidence needs further validation due to the high heterogeneity in cross-sectional studies and the absence of statistical significance in cohort studies. As some inherent limitations in the original studies were observed, we recommend that important potential confounding factors, such as physical activity, should be taken into account (e.g., by statistical adjustment) in further studies. The effect of fiber types on the risk of MetS is also an interesting subject for further investigation. Overall, our meta-analysis suggests that more well-designed prospective studies are needed to confirm the relationship between intake of dietary fiber and risk of MetS.

## Figures and Tables

**Table 1:** Characteristics of published cross-sectional studies on fiber consumption and metabolic syndrome (MetS).

Study	Country	No. of Participants	Type of Fiber	Dietary Assessment	Comparison	OR (95% CI)	Variables Accounted for	Score
McKeown, 2004 [40]	USA	2834	Dietary fiber	FFQ	>22.2 vs. <13.5 g/day	ATP-III: 0.73 (0.51–1.03)	Sex, age, cigarette dose, total energy intake, alcohol intake, percentage saturated fat, percentage polyunsaturated fat, multivitamin use and physical activity	8
Bo, 2006 [41]	Italy	1653	Dietary fiber	FFQ	27.8 (22.6–102.7) vs. 13.2 (3.4–16.4) g/day	ATP-III: 0.49 (0.29–0.83)	Age, sex, BMI, smoking, alcohol intake, level of physical activity, dietary intake of total calories, total percentage of fat, intake of magnesium	8
Steemburgo, 2009 [44]	Brazil	214	Soluble fiberTotal fiber	3-day weighed-diet record	6.3 ± 3.0 vs. 5.2 ± 2.1 g/day20.3 ± 7.8 vs. 16.3 ± 7.0 g/day	IDF: 0.86 (0.74–0.98)IDF: 0.94 (0.90–0.95)	Gender, duration of T2DM and total energy intake	8
Cabello-Saavedra, 2010 [28]	Spain	967	Dietary fiber	FFQ	>26 vs. <18 g/day	ATP-III: 0.55 (0.35–0.86)IDF:0.60 (0.38–0.94)	Age, sex, total energy intake, T2DM, smoking, alcohol intake, education level, marital status and physical activity	9
Kouki, 2011 [31]	Finland	1334	Dietary fiber	4-day food record	Women: 14.6 ± 3.9 vs. 14.1 ± 3.5 g/1000 kcalMen: 13.3 ± 4.0 vs. 12.5 ± 4.3 g/1000 kcal	ATP-III: 0.64 (0.30–1.33)ATP-III: 0.88 (0.44–1.67)	Age, alcohol consumption, smoking, education and VO_2_max	8
Hosseinpour-Niazi, 2011 [42]	Iran	2457	Dietary fiber	FFQ	≥20.7 vs. ≤11.5 g/day	ATP-III: 0.53 (0.39–0.74)	Age, gender, physical activity, smoking status, total energy intake, percentage energy from carbohydrate, fat, saturated fatty acid, cholesterol, magnesium and BMI	8
Silva, 2011 [39]	Brazil	175	Dietary fiber	3-day weighed-diet record	>18.6 g vs. ≤18.6 g/day	IDF: 0.57 (0.29–1.15)	Gender and energy	7
De Oliveira, 2012 [30]	Brazil	305	Dietary fiber	24 h dietary recall	≥20 vs. <20 g/day	Modified ATP-III: 0.67 (0.32–1.38)	Gender, age, BMI and TCV	7
Fujii, 2013 [29]	Japan	4399	Dietary fiber	BDHQ	Mean: 7.60 ± 0.03 g/1000 kcal	Harmonized definition: 0.68 (0.49–0.93)	Obesity, age, sex, duration of diabetes, current smoking habits, current drinking habits, total energy intake, fat intake, saturated fatty acid intake, protein intake (only for urinary albumin excretion and eGFR), leisure time physical activity and use of oral hypoglycemic agents or insulin	7
Grooms, 2013 [43]	USA	10,473	Dietary fiber	24 h dietary recall	22.5–147.6 vs. 0.0–8.1 g/day	ATP-III: 0.78 (0.70–0.88)	Age, sex, race, educational attainment, smoking status, total energy intake	8
Franco, 2014 [27]	Spain	1592	Insoluble fiberSoluble fiber	FFQ	20.87–56.04 vs. 3.60–14.2 g/day9.02–18.37 vs. 1.70–6.05 g/day	ATP-III: 0.62 (0.40–0.96)ATP-III: 0.87 (0.55–1.39)	Age, gender, level of studies completed type of work, energy intake, physical activity, smoking status and alcohol intake	8

Abbreviations: ATP-III: Adult Treatment Panel III of the National Cholesterol Education Program; IDF: International Diabetes Federation; FFQ: food frequency questionnaire; BDHQ: brief self—administered diet history questionnaire; T2DM: type 2 diabetes mellitus; OR: odds ratio; CI: confidence interval; BMI: body mass index; TCV: total caloric value; Score was rated using a 11-item checklist that was recommended by Agency for Healthcare Research and Quality.

**Table 2:** Characteristics of published cohort studies on fiber consumption and MetS.

Study	Country	Case/Participants	Years of Follow-Up	Type of Fiber	Dietary Assessment	Comparison	RR (95% CI)	Variables Accounted for	Score
Hosseinpour-Niazi, 2014 [45]	Iran	240/1582	3	Total dietary fiber	FFQ	≥17.5 vs. ≤12.6 g/1000 kcal	ATP-III: 0.86 (0.63–1.21)	Age, gender, physical activity, smoking status, total energy intake, cholesterol, dairy products, meat, poultry, fish, fat intake, protein intake and BMI	8
Noori, 2010 [46]	Iran	62/160 (BMI)	1	Dietary fiber	FFQ	39 ± 10 vs. 13 ± 3 g/day	ATP-III: 0.41 (0.08–0.99)	Age, sex, cigarette smoking, physical activity, dialysis mode and its duration before transplantation, cumulative dose of steroids at 1 year post-transplant, menopausal status, family history of diabetes, stroke, energy intake, BMI and intake of magnesium	7
		58/160 (WC)							
Carnethon, 2004 [47]	USA	575/4192	15	Crude fiber	Diet history	F(6.9–33)/M(8.6–29.8) vs. F(0.3–2.7)/M(0.6–3.6) g/day	ATP-III: 0.89 (0.68–1.16)	Age, race, sex, BMI, education, smoking, drinking, physical activity, and intake of carbohydrate and fat	9

Abbreviations: ATP-III, Adult Treatment Panel III of the National Cholesterol Education Program; IDF, International Diabetes Federation; FFQ, food frequency questionnaire; RR, relative ratio; CI, confidence interval; WC, waist circumference; BMI, body mass index; F, female; M, male; score was rated by using the Newcastle-Ottawa Scale (NOS).

**Table 3:** Results of subgroup analysis stratified by region, MetS assessment criteria and number of cases.

	*N*	OR (95% CI)	*p*	*I*^2^ (%)
Region				
America	4	0.83 (0.71–0.98)	0.007	75.6%
Europe	6	0.64 (0.52–0.78)	0.692	0.0%
Asia	2	0.60 (0.47–0.77)	0.281	14.0%
MetS assessment criteria				
ATP-III	8	0.69 (0.60–0.79)	0.234	24.4%
IDF	3	0.75 (0.52–1.09)	0.056	65.4%
Number of cases				
≤300	5	0.94 (0.91–0.96)	0.420	0.0%
>300	7	0.68 (0.59–0.78)	0.175	33.1%

Abbreviations: *N*: number of studies; CI: confidence interval; NA: not applicable; ATP-III: Adult Treatment Panel III of the National Cholesterol Education Program; IDF: International Diabetes Federation; OR: odds ratio; *p* value for heterogeneity among studies; *I*^2^, the variation in ES attributable to heterogeneity. America, studies from the U.S. and Brazil; Europe, studies from Finland and Spain; Asia, studies from Iran and Japan.

## Appendix A

**Table A1:** Database search strategies.

Database	Search Strategy
PubMed	Input query:(fiber OR fibre OR food component) AND (metabolic syndrome OR insulin resistance syndrome) AND (cohort OR prospective OR follow-up OR incidence OR cross-sectional OR case-control)PubMed query translation:((“dietary fiber”[MeSH Terms] OR (“dietary”[All Fields] AND “fiber”[All Fields]) OR “dietary fiber”[All Fields] OR “fiber”[All Fields]) OR (“dietary fiber”[MeSH Terms] OR (“dietary”[All Fields] AND “fiber”[All Fields]) OR “dietary fiber”[All Fields] OR “fibre”[All Fields]) OR ((“food”[MeSH Terms] OR “food”[All Fields]) AND component[All Fields])) AND ((“metabolic syndrome”[MeSH Terms] OR (“metabolic”[All Fields] AND “syndrome”[All Fields]) OR “metabolic syndrome”[All Fields]) OR (“metabolic syndrome”[MeSH Terms] OR (“metabolic”[All Fields] AND “syndrome”[All Fields]) OR “metabolic syndrome”[All Fields] OR (“insulin”[All Fields] AND “resistance”[All Fields] AND “syndrome”[All Fields]) OR “insulin resistance syndrome”[All Fields] OR “insulin resistance”[MeSH Terms] OR (“insulin”[All Fields] AND “resistance”[All Fields]) OR “insulin resistance”[All Fields] OR (“insulin”[All Fields] AND “resistance”[All Fields] AND “syndrome”[All Fields]))) AND ((“cohort studies”[MeSH Terms] OR (“cohort”[All Fields] AND “studies”[All Fields]) OR “cohort studies”[All Fields] OR “cohort”[All Fields]) OR (“longitudinal studies”[MeSH Terms] OR (“longitudinal”[All Fields] AND “studies”[All Fields]) OR “longitudinal studies”[All Fields] OR “prospective”[All Fields]) OR follow-up[All Fields] OR (“epidemiology”[Subheading] OR “epidemiology”[All Fields] OR “incidence”[All Fields] OR “incidence”[MeSH Terms]) OR (“case-control studies”[MeSH Terms] OR (“case-control”[All Fields] AND “studies”[All Fields]) OR “case-control studies”[All Fields] OR (“case”[All Fields] AND “control”[All Fields]) OR “case control”[All Fields]) OR (“cross-sectional studies”[MeSH Terms] OR (“cross-sectional”[All Fields] AND “studies”[All Fields]) OR “cross-sectional studies”[All Fields] OR (“cross”[All Fields] AND “sectional”[All Fields]) OR “cross sectional”[All Fields]))Results: 627
Web of Science	Search details:TS = ((“fiber” OR “fibre” OR food component) AND (“metabolic syndrome” OR insulin resistance syndrome) AND (“cohort” OR “prospective” OR “follow-up”OR “incidence” OR “cross-sectional” OR “case-control”))Refined by: [excluding] DOCUMENT TYPES: (EDITORIAL OR LETTER OR MEETING OR BOOK OR REVIEW)Timespan: All years.Search language = AutoResults: 723

Search date: 28 November 2017.
